# A GH115 α-glucuronidase from *Schizophyllum commune* contributes to the synergistic enzymatic deconstruction of softwood glucuronoarabinoxylan

**DOI:** 10.1186/s13068-015-0417-6

**Published:** 2016-01-04

**Authors:** Lauren S. McKee, Hampus Sunner, George E. Anasontzis, Guillermo Toriz, Paul Gatenholm, Vincent Bulone, Francisco Vilaplana, Lisbeth Olsson

**Affiliations:** Wallenberg Wood Science Centre, Division of Glycoscience, School of Biotechnology, KTH Royal Institute of Technology, AlbaNova University Centre, 106 91 Stockholm, Sweden; Wallenberg Wood Science Centre, Division of Industrial Biotechnology, Department of Biology and Biological Engineering, Chalmers University of Technology, 412 96 Gothenburg, Sweden; Wallenberg Wood Science Centre, Department of Chemistry and Chemical Engineering, Chalmers University of Technology, 412 96 Gothenburg, Sweden; Department of Wood, Cellulose and Paper Research, University of Guadalajara, Guadalajara, Mexico; ARC Centre of Excellence in Plant Cell Walls and School of Agriculture, Food and Wine, The University of Adelaide, Waite Campus, Urrbrae, SA 5064 Australia

**Keywords:** Lignocellulosic biomass, Glucuronoarabinoxylan, Glycoside hydrolases (GH), α-Glucuronidase, Agu115

## Abstract

**Background:**

Lignocellulosic biomass from softwood represents a valuable resource for the production of biofuels and bio-based materials as alternatives to traditional pulp and paper products. Hemicelluloses constitute an extremely heterogeneous fraction of the plant cell wall, as their molecular structures involve multiple monosaccharide components, glycosidic linkages, and decoration patterns. The complete enzymatic hydrolysis of wood hemicelluloses into monosaccharides is therefore a complex biochemical process that requires the activities of multiple degradative enzymes with complementary activities tailored to the structural features of a particular substrate. Glucuronoarabinoxylan (GAX) is a major hemicellulose component in softwood, and its structural complexity requires more enzyme specificities to achieve complete hydrolysis compared to glucuronoxylans from hardwood and arabinoxylans from grasses.

**Results:**

We report the characterisation of a recombinant α-glucuronidase (Agu115) from *Schizophyllum commune* capable of removing (4-*O*-methyl)-glucuronic acid ((Me)GlcA) residues from polymeric and oligomeric xylan. The enzyme is required for the complete deconstruction of spruce glucuronoarabinoxylan (GAX) and acts synergistically with other xylan-degrading enzymes, specifically a xylanase (Xyn10C), an α-l-arabinofuranosidase (AbfA), and a β-xylosidase (XynB). Each enzyme in this mixture showed varying degrees of potentiation by the other activities, likely due to increased physical access to their respective target monosaccharides. The *exo*-acting Agu115 and AbfA were unable to remove all of their respective target side chain decorations from GAX, but their specific activity was significantly boosted by the addition of the *endo*-Xyn10C xylanase. We demonstrate that the proposed enzymatic cocktail (Agu115 with AbfA, Xyn10C and XynB) achieved almost complete conversion of GAX to arabinofuranose (Ara*f*), xylopyranose (Xyl*p*), and MeGlcA monosaccharides. Addition of Agu115 to the enzymatic cocktail contributes specifically to 25 % of the conversion. However, traces of residual oligosaccharides resistant to this combination of enzymes were still present after deconstruction, due to steric hindrances to enzyme access to the substrate.

**Conclusions:**

Our GH115 α-glucuronidase is capable of finely tailoring the molecular structure of softwood GAX, and contributes to the almost complete saccharification of GAX in synergy with other *exo*- and *endo*-xylan-acting enzymes. This has great relevance for the cost-efficient production of biofuels from softwood lignocellulose.

**Electronic supplementary material:**

The online version of this article (doi:10.1186/s13068-015-0417-6) contains supplementary material, which is available to authorized users.

## Background

Softwoods represent a potentially valuable renewable resource for the production of biofuels and biomaterials, in addition to their traditional applications in the paper and pulping sectors. In Nordic countries, Norway spruce (*Picea abies*) represents the main source of softwood for the forest industry and is one of the likely lignocellulosic raw materials for second-generation biorefineries. Softwood cell walls primarily comprise cellulose microfibrils enmeshed in a complex matrix of interconnected hemicelluloses and lignin. Although the role of hemicelluloses in secondary cell walls is not fully understood, significant progress has been made in recent years on their heterogeneous molecular structures and how these features modulate hemicellulose interactions with cellulose and lignin [1–3]. The recalcitrance of plant biomass to fractionation and hydrolysis is attributed to the complexity and tight packing of plant cell wall polymers, which interact through a number of cohesive intermolecular forces [4, 5]. Consequently, the biochemical deconstruction of plant cell wall-derived carbohydrates into fermentable sugars, known as a “sugar platform,” is a challenge in the production of biofuel from wood. However, an increased understanding of how hydrolytic enzymes act on these structures will pave the way to more efficient decomposition and hydrolysis, and is therefore an important step towards the realisation of softwood biorefineries.

**Fig. 1:**
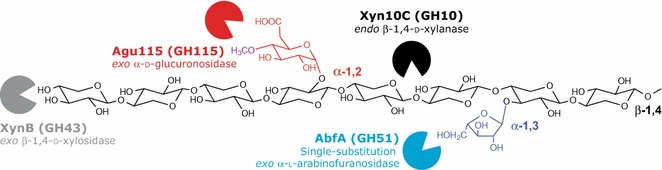
Structure of spruce glucuronoarabinoxylan and sites of action of specific hydrolytic enzymes. Spruce GAX consists of a backbone of β-1,4-linked Xyl*p* residues substituted in places by α-1,3-linked Ara*f* units and α-1,2 linked 4-*O*-MeGlcA. Several enzyme activities are required for the degradation of this highly decorated polymer

Glucuronoarabinoxylan (GAX) is one of the most abundant hemicelluloses in softwood, representing 8–15 % of the total carbohydrate dry weight depending on the plant species [6]. As such, it is highly relevant as a lignocellulosic substrate in biorefineries. However, GAX and xylans in general can adsorb tightly onto cellulose microfibrils and are covalently linked to lignins, making them a relatively inaccessible resource [3, 7, 8]. As shown in Fig. 1, spruce GAX is a highly decorated polysaccharide and is in fact slightly more complex than its hardwood glucuronoxylan (GX) counterpart. GAX consists of a backbone of β-1,4-linked xylopyranosyl units (Xyl*p*), which can be substituted by α-1,3-linked arabinofuranosyl (Ara*f*) residues, and by 4-*O*-methylglucuronic acid (MeGlcA) via α-1,2 linkages. The average degrees of substitution of extracted spruce GAX by Ara*f* and MeGlcA are 7 and 14 %, respectively [9].

The complex structures of polysaccharides such as GAX present significant challenges to saccharification because they require multiple enzyme activities to be fully hydrolysed. The action of glycoside hydrolases (GHs) is typically reduced or inhibited by non-target structural features of polysaccharide substrates, such as the presence and pattern of substitutions along the main chain and the degree of polymerisation of the polymer. For instance, many *endo*-enzymes that cleave polysaccharide backbones are inhibited by the presence of side chains, whereas *exo*-acting enzymes, which remove side chains, are in some cases significantly more active on short-chain oligomeric substrates than on polymers. Thus, hydrolysis to completion is most likely obtained with synergistic combinations of complementary *endo*- and *exo*-enzymes.

Enzymatic removal of the glucuronic acid (GlcA) substituents in spruce GAX has traditionally been a particular stumbling block to fully hydrolyse the polymer. Until recently, all known α-1,2-glucuronidases belonged to GH family 67 (http://www.cazy.org, [10]) and were active only on short xylan oligosaccharides that carry the (Me)GlcA substitution at their reducing ends [11, 12]. The establishment of GH family 115 revealed a group of enzymes that are capable of removing the MeGlcA residues from non-terminal positions of both xylan oligosaccharides and high-molecular weight xylans [13, 14]. Inspired by this observation, we have undertaken the characterisation of a GH115 enzyme from *Schizophyllum commune*, Agu115 [13], and evaluated its potential for the complete hydrolysis of spruce GAX in concert with other xylan-degrading enzymes.

This work is complementary to previous studies on the enzymatic deconstruction of arabinoxylans from cereals. These investigations were based on the use of a combination of *exo*-acting α-l-arabinofuranosidases specific for the single and double Ara*f* substitutions together with *endo*-xylanases and *exo*-β-d-xylosidases targeted to the backbone of the polymer [15, 16]. Here, we analyse and report the synergistic action of Agu115 in combination with commercial enzymes involved in degradation of xylan substituted with both arabinose and (Me)GlcA, namely an *endo*-xylanase (Xyn10C), an α-l-arabinofuranodsidase (AbfA) and a β-xylosidase (XynB). Our data provide further insight into the specificity of Agu115 and a deeper understanding of the enzymatic pre-treatments required for the full degradation of GAX. The results are of high relevance for the implementation of processes to efficiently hydrolyse softwood lignocellulose and biomass conversion in second-generation biorefineries. This study also contributes to our understanding of the potential of Agu115 alone or in combination with AbfA for the topo-enzymatic modification of spruce GAX, in order to tailor the substitution degree of this hemicellulose for material applications.

## Results and discussion

Complex polysaccharides derived from plant biomass, such as softwood GAX, require the concerted action of multiple GHs for complete degradation. Here, we have evaluated the capacity of several enzyme mixtures for GAX hydrolysis. Three GHs were tested against the substrate, in combination with one or both of the other enzymes under analysis. The α-glucuronidase Agu115 was selected due to its recorded action on both oligomeric and polymeric GAX/GX [13]. Initial rate kinetics are presented for Agu115 on native and pre-treated GAX substrates. The other enzymes utilised are all commercially available and have been previously characterised. The α-l-arabinofuranosidase AbfA was included in the study to remove single Ara*f* substitutions from the polymeric substrate [17]. Preliminary tests with an Ara*f*ase specific for removing Ara*f* residues from doubly substituted Xyl*p* sugars [15, 18] indicated that the double substitution structure was not present in this substrate, and it was therefore not considered in further experiments. The *endo*-xylanase Xyn10C was selected for its capacity to degrade highly substituted xylan substrates [19–21]. Finally, the β-xylosidase XynB was added in order to achieve the highest saccharification possible of the spruce GAX substrate [22]. Reaction products from single- and multi-enzyme end-point reactions were identified and quantified, to determine the effect of the combined application of multiple enzymes on product release. The structural profile of the released oligosaccharides after GAX treatment by each enzyme combination was analysed to delineate the structural basis of the limits of each enzyme activity.

### Investigating the specificity of Agu115

The GH115 enzyme utilised in this study, Agu115, is a relatively novel biotechnological tool that has the advantage over the commercially available GH67 α-glucuronidases of being able to remove the (Me)GlcA residues from both inner and terminal substituted xylosyl units in GAX. Instead, GH67 enzymes act exclusively on substitutions carried by terminal xylose residues and thus have detectable activity on oligosaccharides only [12, 23]. Agu115 was initially purified from a secretome produced by the fungus *S. commune* grown on wheat bran [24], as part of a secreted enzyme mixture that also included *endo*-xylanase activity. The enzyme was biochemically characterised as an α-glucuronidase able to act on polymeric xylan. The protein was sequenced, allowing the gene to be identified and cloned for production of recombinant protein in a follow-up paper [13], which characterised the reaction products of the enzyme acting on decorated xylotetraose, and classified the enzyme as belonging to the newly founded glycoside hydrolase family 115. As the (Me)GlcA structure has been a particular impediment to the full hydrolysis of GAX polysaccharides, we feel that this new enzymatic activity warrants further investigation, in light of its potentially significant impact.

**Fig. 2:**
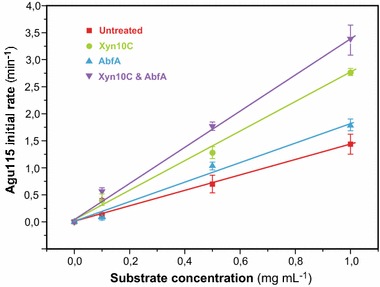
Initial rate analysis for Agu115 on native and enzyme-treated forms of GAX. Specific activity (µmol min^−1^ mg^−1^) for Agu115 acting on these substrates is as follows; GAX: 20.8; GAX pretreated by Xyn10C: 48.8; GAX pretreated by AbfA: 28.9; GAX pretreated by both Xyn10C and AbfA: 52.9. Initial rate of Agu115 activity increases if the GAX is pre-treated by AbfA, Xyn10C, or both

Although Agu115 is active on both oligo- and polymeric GAX, our data show that it does act preferentially on shorter GAX chains, as evidenced by the increase in initial reaction rate of Agu115 following GAX pre-treatment by the *endo*-xylanase Xyn10C, (Fig. 2) and the increased total release of MeGlcA when the substrate is co-incubated with Xyn10C (Table 1). Co-incubation of the polysaccharide substrate with AbfA, Xyn10C, or both, allows Agu115 to release respectively 1.2, 10, and 10.4-fold increased amounts of MeGlcA, compared to Agu115 alone (Table 1). This is comparable to the increases in specific activity and initial reaction rate of Agu115 following equivalent conditions of pre-treatment (Fig. 2 and corresponding legend). The measured specific activity of the enzyme more than doubles (increase of 134 %) if the substrate is pre-treated with Xyn10C, while pre-treatment with AbfA leads to a 39 % increase in activity. Pre-incubation with both AbfA and Xyn10C enzymes has an additive effect, with the resulting observed specific activity of Agu115 increasing by 154 % compared to that measured from the untreated GAX polymer. This potentiation of Agu115 by the other enzyme activities can be explained by the cleavage of the xylan backbone and/or removal of Ara*f* substitutions allowing Agu115 to gain access to more MeGlcA residues more quickly, leading to an overall increased reaction rate. The importance of the removal of MeGlcA substitutions to the overall saccharification of GAX is underlined by the difference in the yield of xylose released by the full cocktail, compared to the cocktail lacking Agu115 (Table 1).

**Table 1:** Mono- and oligosaccharide release (as % in weight of the dry substrate) by the different enzymatic mixtures

Enzyme combination	Ara (%)	Xyl (%)	X^2^ (%)	(Me)GlcA (%)
Blank	nd	1.4 (0.1)	nd	nd
Agu115	nd	1.4 (0.1)	0.1 (0.0)	1.2 (0.1)
AbfA	0.9 (0.1)	1.4 (0.3)	nd	nd
Xyn10C	0.1 (0.0)	8.9 (0.9)	5.5 (0.6)	nd
Agu115 + AbfA	1.4 (0.1)	1.4 (0.0)	0.1 (0.0)	1.5 (0.0)
Agu115 + Xyn10C	0.1 (0.0)	11.9 (0.8)	25.2 (2.4)	12.3 (1.3)
AbfA + Xyn10C	5.9 (0.2)	9.9 (0.3)	14.3 (1.1)	nd
Agu115 + AbfA + Xyn10C	6.4 (0.1)	13.7 (0.5)	35.9 (0.9)	12.5 (0.5)
AbfA + Xyn10C + XynB	7.8 (0.3)	53.3 (2.5)	nd	nd
Agu115 + AbfA + Xyn10C + XynB	7.7 (0.0)	61.6 (2.6)	nd	13.8 (1.0)

The mono- and oligosaccharide release is presented here as % in weight of the total dry substrate, using the quantification data presented in the Additional file 1: Figure S4. The standard deviation is given in brackets

*nd* non-detected

Interestingly, appreciable amounts of linear xylooligosaccharides (XOs) not identified in the original substrate were detected after the action of Agu115 as a single enzyme, which indicates preferential cleavage of MeGlcA from decorated oligosaccharides present in the initial substrate (Additional file 1: Figure S1). The combination of Agu115 with the arabinofuranosidase AbfA also led to the release of longer linear XOs (mainly X^5^ and X^6^), again likely arising from decorated oligosaccharides (mUXOs) present in the starting material. Indeed, the GAX substrate is polydisperse and comprises a fraction of residual oligosaccharides with lower molar masses (see Additional file 1: Figure S2).

### Enzymatic conversion of polysaccharide GAX to mono- and oligosaccharides

**Fig. 3:**
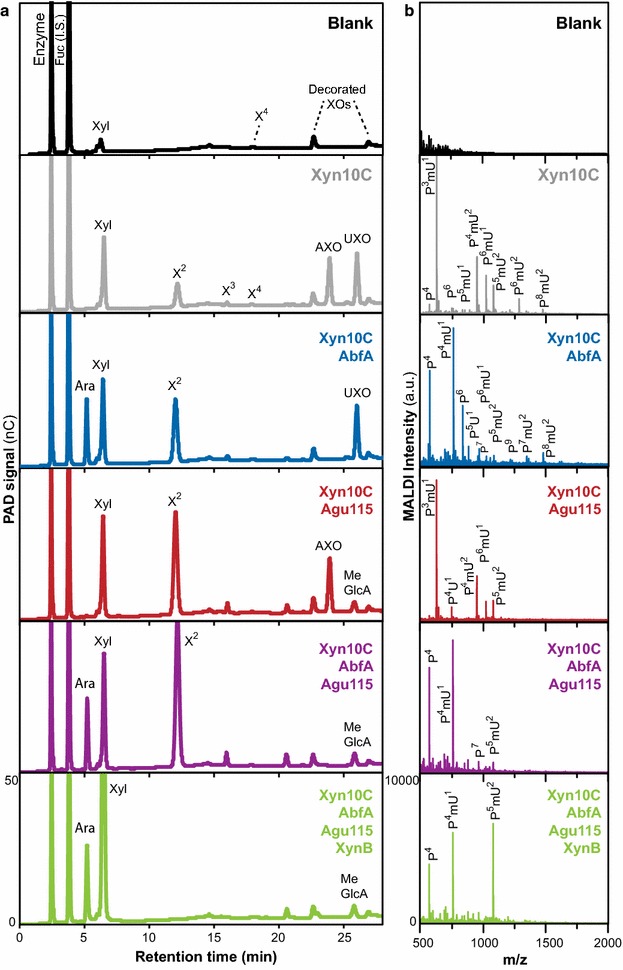
Identification of oligosaccharide reaction products from different enzyme combinations. **a** The chromatographic profiles obtained by HPAEC-PAD enable the identification of the monosaccharides and oligosaccharides released by the different enzyme incubations. Ara, Xyl, and MeGlcA refer to the arabinose, xylose, and 4-*O*-methyl glucuronic acid monosaccharides, respectively. X^2^, X^3^ and X^4^ refer to xylobiose, xylotriose, and xylotetraose, oligosaccharides, respectively, identified by elution of standards. The presence of Ara*f*-decorated xylo-oligosaccharides (AXOs) and MeGlcA-decorated xylo-oligosaccharides (mUXOs) can be specifically assigned in the chromatograms due to the enzymatic activities: AXOs remain after MeGlcA removal by Agu115, while mUXOs remain after Ara*f* removal by AbfA. The scales of the PAD intensities are the same for all the chromatographic profiles. **b** MALDI-ToF–MS assignment of the oligosaccharides released with the different enzyme incubations. *P* pentose (Xyl*p* or Ara*f*), *U* uronic acid (GlcA), mU: 4-*O*-methyl glucuronic acid (4-*O*-MeGlcA). Oligosaccharides that remain after enzyme treatment are named *P* ^*4*^, *P* ^*4*^ *mU* ^*1*^, and *P* ^*5*^ *mU* ^*2*^, where *P* represents a pentose (xylose or arabinose) and *mU* a methylated uronic acid (4-*O*-MeGlcA). Thus, *P* ^*4*^ denotes an oligosaccharide with 4 pentose units, while *P* ^*4*^ *mU* ^*1*^ has an additional MeGlcA substituent. The pentoses (xylose and arabinose) cannot be distinguished by MALDI-ToF–MS due to their isobaric nature. These residual structures are discussed below. The scale of the MALDI intensity is the same for all the MS spectrograms

The production of mono- and oligosaccharides after the combined action of several GHs was followed by both HPAEC-PAD and MALDI-ToF–MS (Fig. 3). This approach allowed qualitative identification of the oligosaccharide products from enzymatic incubations, by correlating molar mass values (MALDI-ToF data) with the patterns of co-elution with appropriate standards on HPAEC-PAD (see “Methods” section). The product profiles of these assays were used to understand the role and contribution of the different enzymes in the hydrolysis of GAX. The HPAEC-PAD spectra presented in Fig. 3 show the release of monosaccharides (Ara*f*, Xyl*p*, and MeGlcA), short linear XOs [ranging from xylobiose (X^2^) up to xylohexaose (X^6^)], and decorated xylooligosaccharides, including arabinose-containing xylooligosaccharides (AXOs) and uronic acid-containing xylooligosaccharides (UXOs), following substrate incubation with Xyn10C and combinations of Agu115, AbfA, and XynB. Additional file 1: Figure S1 shows equivalent data for those reactions lacking the *endo*-enzyme Xyn10C. The release of monosaccharides (Ara*f*, Xyl*p*, and MeGlcA) and short linear XOs was quantified by comparing the HPAEC-PAD response factors with those of known standards. It is worth noting that the MALDI-TOF oligosaccharide profiles do not allow the unequivocal structural determination of the released oligosaccharides, essentially because of the isobaric nature of Xyl*p* and Ara*f*. Thus, the precise location of the substitutions along the backbones of the different AXOs/mUXOs of the same molecular weight remains unknown. However, we can at least estimate the complexity of the decorated oligosaccharides present in each sample using the HPAEC-PAD separation profiles, as decorated oligosaccharides will elute separately from linear oligosaccharide standards of the same mass.

Incubation with the *endo*-xylanase Xyn10C alone releases a complex mixture of Xyl, short linear xylooligosaccharides (XOs) and decorated xylooligosaccharides (both AXOs and UXOs), ranging between four and ten sugar units, as can be observed both in the HPAEC chromatographic profile and the MALDI mass spectrum (Fig. 3). As would be expected, the general trend upon addition of the other enzymes is a decreased complexity of the oligosaccharide profiles and a reduced abundance of the longest oligosaccharides, with a higher abundance of released monosaccharides (Fig. 3; Table 1). Indeed, when AbfA is added to Xyn10C, a reduction in the number and concentration of AXOs allows the identification of the UXO peaks in the chromatograms. Similarly, the combination of Agu115 and Xyn10C should hydrolyse most of the UXOs and allow the identification of the peaks corresponding to the AXOs in the chromatograms. When the combination of all xylan-degrading enzymes was added to the GAX polysaccharide, mainly monosaccharides (Ara, Xyl, and MeGlcA) were observed in the HPAEC profile. Interestingly, several decorated XOs could still be observed in the corresponding MALDI spectra, indicating the presence of residual oligosaccharides that cannot effectively be cleaved to monosaccharides by the enzymatic action. The nature of these recalcitrant oligosaccharides is discussed below.

Table 1 shows the amount of neutral sugars (Ara*f* and Xyl*p*), uronic acids (MeGlcA), and linear XOs (X^2^) released by each enzymatic combination. It is clear from these data that there is a great deal of potentiation between the enzymes (discussed below), and that the activity of the Agu115 α-glucuronidase in particular is boosted by the concomitant action of the other enzymes, mostly by the Xyn10C xylanase.

**Fig. 4:**
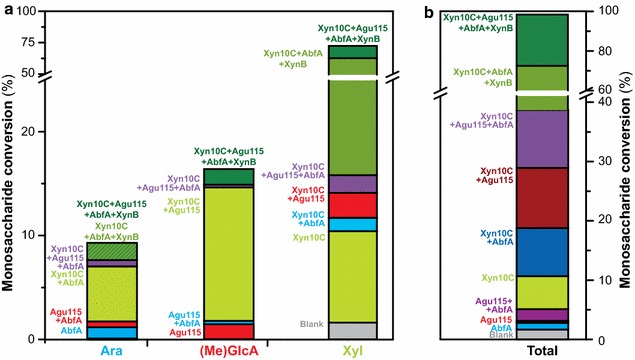
Monosaccharide conversion by the synergistic combination of glycoside hydrolases. **a** Individual monosaccharide conversion (Ara, MeGlcA, Xyl). **b** Total monosaccharide conversion. The *histograms* show the increasing conversion of each and total monosaccharides released by each enzyme or enzyme combination, expressed as the percentage of the theoretical maximal conversion based on the GAX content in the substrate (Additional file 1: Table S1). The *colour key* indicates which enzymes are present in a reaction, showing that there is a cumulative increase in the amount of each sugar released with the inclusion of each additional enzyme into the mixture

The theoretical maximum yield of monosaccharide conversion can be estimated as the glucuronoarabinoxylan content in the substrate (84.3 % in weight), taking into account the presence of Klason lignin and other polysaccharide impurities (mainly pectins) in the substrate preparation (see Additional file 1: Table S1). Figure 4 presents the cumulative effect of each enzyme combination on total monosaccharide conversion based on the theoretical maximum yield. The use of side-chain active enzymes (Agu115 and AbfA) could only release 15 % of the total initial substitutions present in the GAX substrate. The combined action of the backbone cleaving β-xylanase Xyn10C with AbfA and Agu115 led to an almost total hydrolysis of the Ara*f* and MeGlcA substitutions, with X^2^ as the major hydrolysis product. The latter was finally converted fully to Xyl*p* by the inclusion of the β-xylosidase XynB into the reaction mixture, achieving almost complete monosaccharide conversion of the GAX. The influence of the MeGlcA substitutions to the overall monosaccharide conversion efficiency is significant. Indeed, the enzyme cocktail lacking Agu115 (comprising AbfA + Xyn10C + XynB), which is of course incapable of cleaving any MeGlcA substitutions, is also unable to hydrolyse 14 % of the total Xyl*p* present in the GAX substrate. Overall, Agu115 contributes to an increase in conversion corresponding to 25 % of the total maximum yield when added to the enzymatic cocktail. This enzyme cocktail optimised for maximum monosaccharide release, including Agu115 in combination with AbfA, Xyn10C and XynB, hydrolysed 83.1 % (±2.6) of the total dry substrate, which corresponds with 98.6 % (±3.1) conversion of the GAX. Consequently, a complete degradation of the polysaccharide within the experimental margins of error for quantification could be assumed. The remaining GAX corresponds to the apparently recalcitrant oligosaccharides shown in Fig. 3, and discussed below.

Although our enzyme cocktail could achieve almost complete hydrolysis of the GAX substrate, minor amounts of certain residual oligosaccharides were detected by MALDI-ToF–MS (Fig. 3). It was not possible to quantify exactly within the experimental error the amount of these residual oligosaccharides due to the statistical deviation of the quantification procedure, but they can be estimated as 1.4 % of the theoretical yield. We have named these oligosaccharides *P*^*4*^, *P*^*4*^*mU*^*1*^, and *P*^*5*^*mU*^*2*^, where *P* represents a pentose (xylose or arabinose) and *mU* a methylated uronic acid (4-*O*-MeGlcA). Thus, *P*^*4*^ denotes an oligosaccharide with four pentose units, while *P*^*4*^*mU*^*1*^ has an additional MeGlcA substituent. These recalcitrant oligosaccharides likely arise from short regions of the GAX substrate with a particularly high density of substitutions, which cannot be fully hydrolysed by the combined hydrolytic activities utilised in this enzyme mixture, due to steric hindrances. From the conversion data it seems that these oligosaccharides are heavily substituted with (Me)GlcA, but the absence of Ara*f* substitutions cannot be entirely ruled out. Additionally, our data do not provide information on the precise placing of substitutions to the xylan backbone, but do indicate that close packing of MeGlcA and Ara*f* occurs in specific clusters. There is evidence for xylans having so-called ‘major’ and ‘minor’ domains, regions with respectively more and less densely packed GlcA and other substitutions [1, 2]. Preliminary tests using a specific Ara*f*ase [15, 18] indicated that there were no Xyl*p* residues doubly substituted with two Ara*f* decorations, but it is currently unknown whether a backbone Xyl*p* can be substituted with both an Ara*f* and a (Me)GlcA in GAX. A close analysis of these residual oligosaccharides is currently underway, and will shed further light on the spacing of Ara*f* and (Me)GlcA decorations in softwood GAX.

### Potentiation of enzyme activities

The use of Agu115 as a single enzyme on the untreated GAX led to the release of MeGlcA representing 1.2 % by weight of the total dry substrate (8.5 % total MeGlcA content), (Table 1). Co-incubation of Agu115 with AbfA slightly increased the amount of MeGlcA released (1.5 % total dry substrate, 11 % total MeGlcA). Similarly, the combined action of Agu115 and AbfA led to a slightly higher release of Ara*f* compared to the action of AbfA alone (0.9 vs 1.4 % total dry substrate, or 12 vs 18 % total Ara*f*). These data indicate that the presence of non-target substitutions on the xylan backbone in close proximity to the respective monosaccharide substrates of each *exo*-enzyme might reduce the efficacy of those enzymes. This effect is presumably due to steric hindrance, with the close proximity of non-target substitutions hampering an enzyme from gaining physical access to its specific target residue.

For maximum monosaccharide yield, effective combinations of enzymes were achieved by pairing a GH acting on monosaccharide substitutions with the backbone cleaving enzyme Xyn10C. As can be observed from the chromatographic profiles (Fig. 3), the *endo*-xylanase Xyn10C used as a single enzyme released a rather complex mixture of linear and substituted XOs. Xyl and X^2^ were the major products (approximately 25 % of total products), along with smaller amounts of X^3^ and X^4^, and several longer, decorated oligosaccharides (AXO and mUXOs) of 5–10 sugar residues, due to the Ara*f* and MeGlcA decorations on the xylan backbone that prevent the xylanase from creating shorter reaction products (Fig. 3). The yield of Xyl from the *endo*-acting enzyme Xyn10C action represented 14 % of the total Xyl in the substrate. Xyn10C was potentiated by both of the enzymes that remove monosaccharide decorations from the substrate. The removal of side chains by Agu115 or AbfA enhanced the ability of Xyn10C to access the xylan backbone, leading to a fivefold and a 2.5-fold increase in the yield of X^2^, respectively (Additional file 1: Figure S4; Table 1), and a concomitant increase in the Xyl yield as well. The enhanced boost in the yield of X^2^ caused by Agu115 compared to AbfA tallies again with the compositional data showing that MeGlcA is a more common substituent in GAX compared to Ara*f*: Agu115 action removed more obstacles to Xyn10C activity than did AbfA. In terms of enzyme potentiation, the most striking activity boost is seen in the release of MeGlcA by Agu115, which is dramatically increased by the synergistic action of Xyn10C, with a tenfold increase compared to Agu115 acting alone (Table 1). This indicates that Agu115 preferentially releases MeGlcA from oligosaccharides rather than from polymeric GAX (echoed in the Agu115 rate analysis, discussed above). The influence of Xyn10C on the release of Ara*f* by AbfA is also notable. However, in this case, a lower (sixfold) increase in released sugar is observed compared to Agu115, suggesting that AbfA is also somewhat more active on shorter xylan chains. A similar observation has previously been made for another GH51 Ara*f*ase [25]. This boost in side chain release by both Agu115 and AbfA in the presence of Xyn10C might be related to an increase in substrate accessibility arising from enhanced solubility and/or changes in the macromolecular conformation of the GAX following Xyn10C action, rather than being related to the actual specificity of the enzymes. Cleavage of the GAX backbone by the Xyn10C might have reduced substrate aggregation, allowing Agu115 access to MeGlcA substituents potentially buried within an aggregated GAX particle. However, dynamic light scattering (DLS) analysis of the substrate verified that under the experimental conditions utilised here, the GAX substrate was molecularly soluble with a negligible number of aggregated molecules (Additional file 1: Figure S3). Therefore, the DLS results support our observation that both Agu115 and AbfA are more active on oligomeric substrates than polymeric ones, although the influence of other conformational or physical features of the substrate, such as an increased molecular flexibility or mobility with reduced molecular weight of GAX after Xyn10C action, cannot be ruled out.

### Tailoring the enzyme cocktail for specific applications

Replacing some of the enzymes used in this cocktail with others of slightly different specificity would likely lead to significant differences in terms of products obtained and yields of hydrolysis. Specifically, the inclusion of a GX-specific xylanase from family GH30, which requires (Me)GlcA substitutions to depolymerise the xylan backbone [26], could have led to very different results. Pre-treatment by a GH30 enzyme such as XynC, which cleaves GX to produce XOs with MeGlcA on the xylose penultimate from the reducing end [27], would lead to targeted depolymerisation of G(A)X to produce MeGlcA substituted XOs which our data suggest are better substrates for Agu115 than polymeric GAX. This could lead to a different level of potentiation of Agu115, perhaps increasing the overall yield of (Me)GlcA. However, co-incubation of Agu115 and XynC would cause competition between the two enzymes for access to areas of the polysaccharide with MeGlcA substitutions, likely decreasing the overall efficiency of the enzyme cocktail for saccharification. However, the effect of Ara*f* substitutions on the activity of this GH30 GX-specific xylanase is not known, so it is difficult to predict the synergistic interactions of such an enzyme with this enzyme cocktail, and it may be that a different population of recalcitrant structures would result upon combination with AbfA and Agu115. Alternatively, using a xylanase from family GH11 instead of Xyn10C would likely have resulted in larger residual oligosaccharide products. It has previously been observed that while the typical end product from total degradation of GX by a GH10 is a substituted X^3^ [19], the end product from a GH11 xylanase is a substituted X^4^ [21, 28, 29]. Different combinations of hydrolytic enzymes will clearly have different levels of efficacy in terms of monosaccharide yield. It is important therefore that enzyme cocktails such as that presented here must be carefully tailored to the specific purpose of polysaccharide degradation by close study of the precise specificities of the enzymes included.

In general, glucose is the most useful and valuable monosaccharide for fermentation processes, as it is the most easily fermented. However, work is ongoing to engineer yeast strains capable of fermenting sugars such as those found in xylan [30–32], and the high monosaccharide conversion yields possible using the enzyme cocktail suggested in the present work are important for the utilisation of xylose-rich lignocellulosic streams. It is noteworthy that the enzymatic deconstruction of GAX demonstrated here took place at 40 °C, close to the optimal growth temperature of the industrially important yeast *Saccharomyces cerevisiae*. The moderate thermal requirements of the hydrolysis we describe avoid the need for costly high-temperature processes, and may allow for simultaneous saccharification and fermentation (SSF) processes, if pentose metabolising yeast strains are utilised. The residual oligosaccharides in our samples represent a very minor proportion of the overall GAX, and consequently allow for efficient use of a woody biomass-based sugar platform. Although it has been shown that xylooligosaccharides can have a strong inhibitory effect on the enzymatic degradation of cellulose via competitive non-productive binding to industrial cellulases such as *Tr*Cel7A [33], they are a very minor factor in our hydrolytic system. An efficient hydrolysis of xylan containing hemicellulose as demonstrated here is instrumental for material and cost-efficient lignocellulose-based processes.

For utilisation of lignocellulose-derived sugars for fermentation processes, the wood carbohydrate polymers cellulose and hemicelluloses will be hydrolysed to their component monosaccharides. A typical process will involve pre-treatment steps, where the structure of lignocellulose is opened up, and some polymeric material is solubilised. This is followed by a hydrolysis step, often performed by enzymes, to deconstruct these polysaccharides. Many challenges may arise during the enzymatic hydrolysis, related to the structural features of the lignocellulosic substrates as well as enzymatic factors, such as substrate specificity and optimal conditions for activity, which can reduce the efficiency of this process. In general, to offer good water economy, the process requires efficient processing, high product concentrations to reduce the need for down-stream processing, and high substrate concentration [34], which also introduces a number of challenges. Up-scaling of the saccharification of GAX from softwood biomass requires optimisation of the substrate concentration and enzyme loading conditions for maximum release of fermentable sugars. Previous studies on the effect of substrate concentration on simultaneous saccharification and fermentation (SSF) for the production of ethanol from lignocellulosic biomass make recommendations of substrate loading within 2–15 % (*w/v*) or even solid contents up to 30 % (*w/v*) [35–37], values which are an order of magnitude higher than those employed in the present study. Such high substrate loading conditions could affect the aggregation state of the substrate and the substrate accessibility of the enzymes in this cocktail, thereby reducing the yields of fermentable sugars. However, the use of Xyn10C should reduce the level of GAX aggregation even at high substrate concentrations, to maintain solubility and a high yield of overall conversion. In an industrial setting, however, GAX is unlikely to be used as a purified substrate, but will be a component of more complex pre-treated biomass and the physical properties of the substrate will depend on the pretreatment method utilised. This increased complexity will likely introduce other potential aggregation and enzyme accessibility effects, caused by the supramolecular interactions of the GAX with cellulose microfibrils, other hemicelluloses (mainly galactoglucomannan in softwoods), and lignins. The up-scaling of the saccharification of softwood biomass using our enzymatic cocktail, applied in combination with cellulases and other hemicellulases, should be studied separately for a maximal release of fermentable sugars.

The debranching enzymes utilised in this study are able to act on polymeric substrate, whereas other *exo*-acting enzymes are often active only on oligomeric substrate (e.g. the α-1,2-glucuronidases from GH family 67 [11, 12]). However, even the enzymes described here are restricted in their application to polysaccharides, as shown by the significant potentiation of AbfA and Agu115 activities by Xyn10C. This may limit the application of these particular enzymes in certain ways and call for the identification and use of enzymes that act more efficiently on polymeric substrates. On the other hand, the use of the here applied enzymes may also open other less destructive avenues of application, as partial enzymatic degradation of a polysaccharide can be useful in producing specific carbohydrates with tailored structures. Indeed, residual oligosaccharides like those we describe may themselves prove to be of use for some applications such as, for example, dietary supplements [38–41] or in the development of biochemicals and bio-based materials [42, 43]. For applications utilising GAX polysaccharide, the enzymes AbfA and Agu115 discussed here are of use in modifying the degree of substitution of GAX, with impacts on the physical properties of the polysaccharide, including solubility and rheology. Our results show that these enzymes are not capable of producing linear xylan from a heavily decorated substrate, as even the combined action of both Agu115 and AbfA is not enough to achieve full removal of substitutions from the polymeric GAX backbone. This has implications for the use of these enzymes in the deconstruction of woody biomass, as it has been reported that lowly substituted xylan shows enhanced adhesion onto cellulose microfibrils [3, 7, 8] and that linear xylan is able to form ordered crystalline structures [3, 44]. This observation also impacts the potential exploitation of such enzymes for the design of tailored xylan-based materials, as they will be most effectively employed for modification of oligomeric GAX, which will have different biotechnological applications than polymeric GAX.

## Conclusions

We present here a tailored enzymatic cocktail to achieve efficient hydrolysis of softwood xylans, which is instrumental for material- and cost-efficient processes for the generation of biofuels from lignocellulose-based streams. High yields of monosaccharides were obtained from spruce GAX by hydrolysis with a combination of complementary GH enzymes, and we show how *exo*- and *endo*-acting enzymes work together for the deconstruction of a complex hemicellulose with different decorations along the backbone. Closer study of the activity of each enzyme revealed a high degree of cross-potentiation, with each enzyme creating better substrate access for the others. Although active on the polymeric GAX substrate, the *exo*-acting enzymes Agu115 and AbfA are unable to completely remove all the sugar decorations alone, and require the cooperative action of an *endo*-acting β-xylanase (Xyn10C) for optimised hydrolysis. Certain residual oligosaccharides remained even after prolonged incubation with all of the hydrolytic enzymes tested. These structures likely arise from areas of the GAX substrate with an unusually high density of MeGlcA substitutions, and indeed there is literature indicating that these decorations can cluster on xylan polysaccharides [1]. The results of the present study of cooperative enzymatic activities have important significance for enhancing the release of fermentable sugars and therefore generate an attractive sugar platform from lignocellulosic biomass to produce products such as bioethanol by fermentation, and for the production of hemicellulosic polymers and oligosaccharides with tailored molecular structures for material applications.

## Methods

### Chemicals and enzymes

All chemicals were of analytical grade and purchased from Sigma–Aldrich, except for linear xylooligosaccharide standards of a DP of 2–6, which were from Megazyme. Table 2 describes all enzymes used in this study. All enzymes were purchased from different suppliers, except for Agu115, which was produced recombinantly as follows. The codon optimised Agu115 gene from *S. commune* was cloned by NZYTech (Lisboa, Portugal) into a proprietary vector containing a C-terminal c-myc and His_6_ tag, and its product was expressed in *Komagataella pastoris* (*Pichia pastoris*). The cloning procedure introduced C-terminal c-myc and His_6_ tags in the protein. Recombinant protein production in *P. pastoris* was performed in a fed-batch culture for 5 days at 25 °C, using a starting volume of 10 L in a 20 L Techfors fermentor (Infors HT, Bottmingen, Switzerland) [45]. Expression of the recombinant protein, which was secreted into the growth medium, was induced by the addition of methanol, controlling the feeding rate to maintain a dissolved oxygen tension above 20 %. The culture was centrifuged at 10,000*g* and 4 °C for 10 min to collect the supernatant liquid, which was filtered through a Millistak+^®^ Pod, A1HC with 0.054 m^2^ filtration area, before being concentrated and buffer exchanged into 50 mM sodium acetate, pH 5.7 with ultrafiltration using two serially connected Pellicon^®^ 2 Mini Cassettes of 0.1 m^2^ with a nominal cut-off of 10 kDa (Merck Millipore, Solna, Sweden). Anion exchange chromatography was performed with a HiLoad 16/10 Q-Sepharose HP (GE Healthcare, Uppsala, Sweden) column, equilibrated with 50 mM sodium acetate, pH 5.7. The adsorbed proteins were eluted by the gradual addition of NaCl to the equilibration buffer. Agu115 was eluted at 250 mM NaCl. The fractions with the highest activity were pooled and subjected to subsequent gel filtration on a HiPrep 26/60 Sephacryl S-200 HR column (GE Healthcare, Uppsala, Sweden), using the same equilibration buffer supplemented with 150 mM NaCl. Purifications were performed using an Äkta Purifier FPLC system (GE Life Sciences, Uppsala, Sweden). Fractions were collected and tested for activity using the α-glucuronidase assay kit (K-AGLUA, Megazyme, Ireland). Alduronic acids (a mixture of tri- tetra- and pentasaccharides) were used as the substrate, and the released d-glucuronate was measured with the addition of NAD+ and uronate dehydrogenase by measuring the absorbance of the resulting NADH at 340 nm. The fraction showing the highest level of activity was retained for analysis. The enzyme had been previously characterised to determine its activity and optimal conditions of temperature and pH (Table 2 and [13]).

**Table 2:** Description and properties of the enzymes employed for the deconstruction of softwood xylan

Enzyme	Family	Activity	Source organism	pH_opt_	pH_stab_	*T*_opt_ (°C)	*T*_stab_ (°C)	Supplier
Xyn10C	GH10	*Endo*-β-1,4-xylanase	*Clostridium thermocellum*	5.5	4–11	65	Max 75	NZYtech
XynB	GH43	*Exo*-β-1,4-xylosidase	*Bacillus pumilus*	7.5	NR	35	Max 50	Megazyme
AbfA	GH51	α-l-Arabinofuranosidase	*Aspergillus niger*	4	4–8	40	Max 50	Megazyme
Agu115	GH115	α-Glucuronosidase	*Schizophyllum commune*	5.8	6–8	40	Max 40	Produced in-house

*NR* denotes that this information was not reported by the manufacturer

### GAX substrate

GAX was isolated from Norway spruce wood following an alkaline extraction protocol [9]. In brief, spruce wood meal was delignified by four consecutive additions of sodium chlorite and acetic acid at 70–80 °C every 12 h. Hemicelluloses were successively extracted from the resulting holocellulose with 24 % KOH for 24 h at room temperature and precipitated from the extract with acidic ethanol, filtered and dried. Hemicelluloses were re-dissolved in 10 % KOH at room temperature for about 30 min and the solution was treated with a saturated solution of Ba(OH)_2_. The supernatant containing GAX was mixed with acidic ethanol to precipitate the polymer. The yield was approximately 7 % (*w/w*) of the original spruce softwood raw material.

The GAX substrate contained 5.7 %, *w/w* of Klason lignin that is likely attached covalently to the carbohydrate chains (Additional file 1: Table S1). Preliminary carbohydrate analysis by methanolysis showed the presence of other polysaccharide constituents in the substrate (10 % *w/w*), which could be assigned to galactan, homogalacturonan, and rhamnogalacturonan (Additional file 1: Table S1). The GAX substrate also contained traces of monosaccharides (xylose, approximately 4 mg/g) and oligosaccharides, mainly xylotetraose (approximately 1 mg/g), and other decorated oligosaccharides (Additional file 1: Figure S1). The weight-average molar mass (*M*_w_) of the GAX substrate is 19,640 g mol^−1^, with a dispersity of 1.5 as measured by size-exclusion chromatography (Additional file 1: Figure S2), indicating the presence of a fraction of oligosaccharides with lower molar masses. Prior to enzymatic incubation, the dry substrate was suspended in Milli-Q water and heated to 65 °C with frequent shaking, which provided a clear solution, by visual observation. The molecular solubility of the substrate was verified by dynamic light scattering (DLS), which indicated that the presence of aggregated GAX particles was negligible under our experimental conditions (see Additional file 1: Figure S3).

### Enzymatic hydrolysis of GAX

The GAX substrate was hydrolysed by either one of the enzymes listed in Table 2 or various combinations of these (see Table 1). All reactions were performed on the same batch of GAX in 1.5 mL microcentrifuge tubes (Eppendorf) in 50 mM citrate buffer (pH 6) at 40 °C with shaking (180 rpm) for 16 h, in a total volume of 500 µL. Initial screening experiments confirmed that no additional release of sugars was achieved after 16 h. The mixtures consisted of 1 mg mL^−1^ GAX, 10 μg mL^−1^ fucose (internal standard used for subsequent product analysis) and one or several enzymes at the following concentrations: Agu115 7.9 µM; AbfA 0.45 U mL^−1^ (activity determined on pNP-α-l-Ara*f*); Xyn10C 0.32 U mL^−1^; (activity determined on wheat arabinoxylan); XynB 0.2 U mL^−1^ (activity determined on pNP-β-d-Xyl*p*). After hydrolysis, the samples were boiled, centrifuged at 4 °C, and the supernatant was retained for product analysis. Each enzyme combination was assayed three times to ensure the statistical validity of our characterisation of a relatively heterogeneous polysaccharide.

### Identification and quantification of reaction products

Monosaccharides and oligosaccharides released in the reaction mixtures were identified and quantified using mass spectrometry and ion exchange chromatography. Oligosaccharide products of GAX degradation were identified by MALDI-ToF–MS on an LT3 Plus mass spectrometer (SAI Ltd., UK) operated by the MALDI Mainframe 2, MALDI Control software (version 1.03.51, SAI Ltd., UK). The hydrolysates were desalted using a Hypercarb Hypersep cartridge (Thermo Scientific, Bellefonte, PA, USA) and mixed with a matrix of 2,5-dihydroxybenzoic acid (DHB) [10 mg ml^−1^ in acetone (50 % *v/v*)] directly on the MALDI plate prior to analysis. Products from the hydrolytic reactions were separated and quantified by high-performance anion exchange chromatography with pulsed amperometric detection (HPAEC-PAD) on an ICS3000 system (Dionex, Sunnyvale, CA). The reaction mixtures were diluted in deionised water (1:10 *v/v*) and 10 μL were injected onto a Dionex Carbopac PA1 column maintained at 30 °C at a flow rate of 1 mL min^−1^. Different gradients were employed for the detection and quantification of the hydrolysis products (Solvent A: deionised water; solvent B: 300 mM sodium hydroxide; solvent C: 200 mM sodium hydroxide + 170 mM sodium acetate; solvent D: 1 M sodium acetate). For the analysis of neutral monosaccharides, the column was equilibrated for 7 min with 60 % (*v/v*) solvent B and 40 % (*v/v*) solvent C, ramped to 100 % (*v/v*) solvent A for 1 min, and further equilibrated at 100 % (*v/v*) solvent A for 6 min prior to injection. The samples were eluted over 20 min with 100 % (*v/v*) solvent A, and detected with post-column addition of 0.5 mL min^−1^ of 300 mM sodium hydroxide. For uronic acid analysis, the column was equilibrated with 10 % (*v/v*) solvent B for 5 min prior to injection. The samples were eluted with a 30 min gradient to 10 % (*v/v*) solvent B + 40 % (*v/v*) solvent D. Separation of xylooligosaccharides was performed by equilibrating the column with 33 % (*v/v*) solvent B for 5 min prior to injection. The samples were eluted with a 30 min gradient to 33 % (*v/v*) solvent B + 20 % (*v/v*) solvent D. The different reaction products were quantified by comparing the peak areas of the analytes with those of standards at known concentrations, using a standard curve for calculation. The response factor for MeGlcA was calculated from the analytical response factor for GlcA with the appropriate correction as reported in [46].

### Analysis of Agu115 reaction rates

Kinetic analysis was performed at the optimum conditions of pH and temperature for Agu115, i.e. pH 6.0 and 40 °C (Table 2). In some cases, GAX was pre-treated for 2 h in the conditions described in the previous section (Enzymatic hydrolysis of GAX), with Xyn10C, AbfA, or both of these enzymes, prior to rate analysis. The reactions were stopped by boiling for 5 min, then a brief centrifugation at 10,000 rpm was performed to pellet any precipitated material. The initial rate of reaction of the Agu115 recombinant α-glucuronidase against GAX was determined using the linked uronic acid dehydrogenase assay (K-AGLUA, Megazyme, Ireland). The release of GlcA led to the stoichiometric reduction of NAD+ to NADH, giving an increase in *A*_340_ (ε 6230 M^−1^ cm^−1^ [47]), which was read continuously in real-time using a Cary 300 spectrophotometer. Kinetic experiments were performed in triplicate.

## Additional file

10.1186/s13068-015-0417-6 Composition of the GAX substrate as %wt of the total dry content. **Figure S1.** Identification of mono- and oligosaccharides in the GAX substrate and enzymatic reaction mixtures by HPAEC-PAD: arabinose (Ara), xylose (Xyl), 4-*O*-methyl glucuronic acid (MeGlcA), xylotetraose (X^4^), xylo-oligosaccharides (XOs). **Figure S2.** Molar mass distribution and averages for the GAX substrate. **Figure S3.** DLS analysis of the GAX substrate. **Figure S4.** Quantification of the monosaccharides and linear xylo-oligosaccharides (XOs) released by each enzyme/enzyme combination.

## Abbreviations

GlcA: glucuronic acid
MeGlcA: 4-*O*-methyl glucuronic acid
Xyl*p*/Xyl: xylopyranose
Ara*f*/Ara: arabinofuranose
GH: glycoside hydrolase
Ara*f*ase: α-l-arabinofuranosidase
GAX/GX: glucuronoarabinoxylan/glucuronoxylan
HPAEC-PAD: high-performance anion exchange chromatography with pulsed amperometric detection
MALDI-ToF–MS: matrix-assisted laser desorption/ionisation time-of-flight mass spectrometry
X^2^: xylobiose
X^3^: xylotriose
X^4^: xylotetraose
X^5^: xylopentaose
X^6^: xylohexaose
SSF: simultaneous saccharification and fermentation
