# Supplementary material for: A GH115 α-glucuronidase from Schizophyllum commune contributes to the synergistic enzymatic deconstruction of softwood glucuronoarabinoxylan
Source: Biotechnol Biofuels. 2016 Jan 4;9:2. doi: 10.1186/s13068-015-0417-6 (PMC4700659; doi:10.1186/s13068-015-0417-6)
Supplement: Supplementary file 1 — 10.1186/s13068-015-0417-6 Composition of the GAX substrate as %wt of the total dry content. Figure S1. Identification of mono- and oligosaccharides in the GAX substrate and enzymatic reaction mixtures by HPAEC-PAD: arabinose (Ara), xylose (Xyl), 4-O-methyl glucuronic acid (MeGlcA), xylotetraose (X4), xylo-oligosaccharides (XOs). Figure S2. Molar mass distribution and averages for the GAX substrate. Figure S3. DLS analysis of the GAX substrate. Figure S4. Quantification of the monosaccharides and linear xylo-oligosaccharides (XOs) released by each enzyme/enzyme combination. [file 13068_2015_417_MOESM1_ESM.docx]

**Supplementary Material**

# A GH115 α-Glucuronidase from *Schizophyllum commune* Contributes to the Synergistic Enzymatic Deconstruction of Softwood Glucuronoarabinoxylan

Lauren S. McKee^1^, Hampus Sunner^2^, George E. Anasontzis^2^, Guillermo Toriz^2,3^, Paul Gatenholm^2^, Vincent Bulone^1,4^, Francisco Vilaplana^1*^, Lisbeth Olsson^2*^

## Substrate characterization

### Compositional analysis of the GAX substrate

Klason lignin (acid insoluble lignin) was determined gravimetrically after sulphuric acid hydrolysis of the dried GAX sample, using an adapted TAPPI method [1,2]. In brief, approximately 10 mg of sample was subjected to two-step acid hydrolysis: the first step with 72% H_2_SO_4_ for 1 h at room temperature and a second step with 3% H_2_SO_4_ for 3 h at 100°C. The Klason lignin content was calculated gravimetrically from the non-hydrolysed vacuum filtrate as 5.7% (w/w) of the total GAX dry content (Table S1).

Carbohydrate analysis was performed by methanolysis and subsequent analysis of the released monosaccharides by high-performance anion-exchange chromatography with pulsed amperometric detection (HPAEC-PAD) [3,4]. In brief, 1 mg of the GAX sample was subjected to acid hydrolysis with 2M HCl in methanol at 100°C for 5 h and a subsequent step with trifluoroacetic acid (TFA) 2M at 121°C for 1 h. The hydrolysate was dried and redissolved in deionized water prior to HPAEC-PAD analyses for the quantification of neutral sugars and uronic acids (see main text, Materials and Methods). The samples were analysed in triplicate (Table S1).

The substrate consists of 84.3 % (w/w dry weight) glucuronoarabinoxylan. This can be considered as the maximum theoretical yield that could be achieved by the synergistic combination of the enzymes in the main text. The remaining dry weight may be associated to other polysaccharide impurities (mainly pectins such as galactan, galacturonan and rhamnogalacturonan), and to lignin, which may be covalently bound to the xylan as typically described in the structure of lignocellullosic biomass.

Table S1 Composition of the GAX substrate as %wt of the total dry content.

| **Composition**  (% dry weight) | |
| --- | --- |
| **Lignin** | 5.7 |
| **Carbohydrates** | |
| Fuc | 0.1 (0.0) |
| Ara | 7.8 (0.2) |
| Rha | 0.7 (0.0) |
| Gal | 1.5 (0.0) |
| Glc | 3.3 (0.4) |
| Xyl | 61.9 (1.7) |
| Man | < 0.1 |
| GalA | 4.4 (0.2) |
| GlcA | 0.5 (0.1) |
| MeGlcA | 14.1 (1.1) |

The lignin content was calculated according to the Klason method, whereas the carbohydrate content was determined by methanolysis and HPAEC-PAD quantification. The standard deviation of the carbohydrate analysis is presented between brackets.

### Determination of mono- and oligosaccharides in substrate and enzyme reaction mixtures

The identification and quantification of mono- and linear oligosaccharides in the substrate and enzyme mixtures was performed by high-performance anion-exchange chromatography with pulsed amperometric detection (HPAEC-PAD), as described in the main text (Materials and Methods). The presence of xylose (Xyl, 4 μg mg^-1^ substrate), xylotetraose (X^4^, approximately 1 μg mg^-1^ substrate) and other decorated xylo-oligosaccharides (XOs) can be observed in the substrate (Figure S1). Moreover, the progressive liberation of arabinose (Ara) and 4-O-methyl glucuronic acid (MeGlcA) can be observed by the individual and combined actions of AbfA and Agu115, respectively.


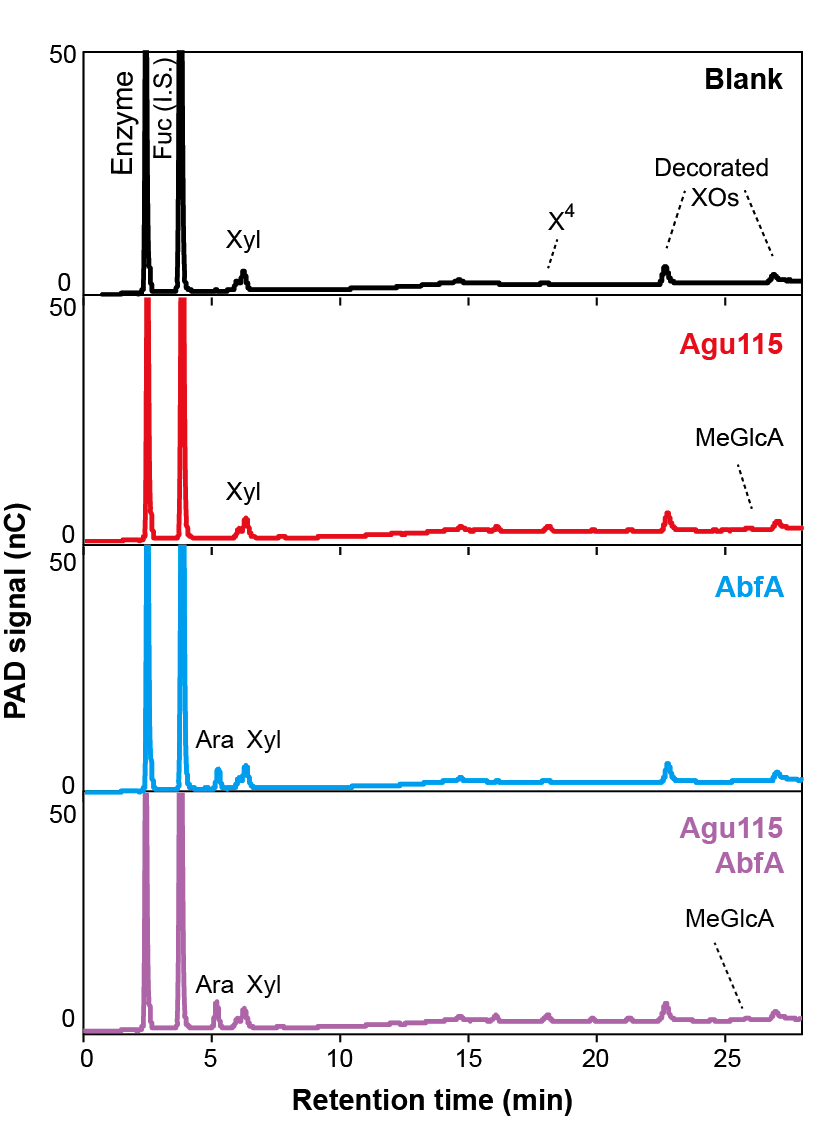


Figure S1 Identification of mono- and oligosaccharides in the GAX substrate and enzymatic reaction mixtures by HPAEC-PAD: arabinose (Ara), xylose (Xyl), 4-O-methyl glucuronic acid (MeGlcA), xylotetraose (X^4^), xylo-oligosaccharides (XOs)

### Molar mass distribution of the GAX substrate

The molar mass distribution and average molar masses for the GAX substrate were determined by size exclusion chromatography (SEC) using a SECcurity 1260 system (Polymer Standard Services, Mainz, Germany) coupled to a refractive index detector at 45°C. Separation was performed with DMSO/LiBr (0.5% w/w) as the mobile phase on a column system consisting of a GRAM PreColumn, 30 and 10000 analytical columns (Polymer Standards Services, Mainz, Germany), with a flow rate of 0.5 mL min^-1^ at 60°C. Relative calibration was performed by the injection of pullulan standards of molar masses in the range of 342 – 760000 g mol^-1^ provided by Polymer Standards Services (PSS, Mainz, Germany). The apparent molar mass distribution of the GAX substrate was determined based on the retention time of the linear pullulan standards.

The GAX substrate shows a monomodal molar mass distribution (Figure S2), with a weight-average molar mass (*M*_w_) of 19640 g mol^-1^ and a fairly narrow polydispersity of 1.5. A significant tail of the distribution can be observed towards the lower molar mass range, which indicates the presence of a small fraction of residual oligosaccharides that consist of 10 – 20 sugar units.


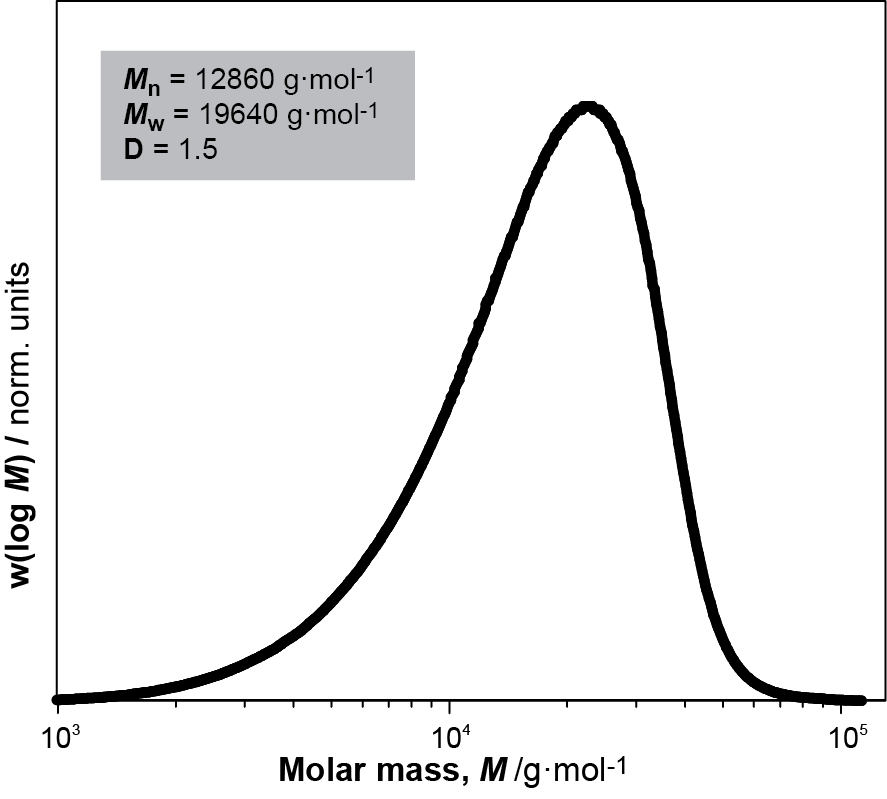


Figure S2 Molar mass distribution and averages for the GAX substrate

### Dynamic Light Scattering (DLS) analysis of the substrate

The molecular solubility and potential aggregation of the GAX substrate were studied by Dynamic Light Scattering (DLS) using a Zetasizer Nano-ZS (Malvern Instruments Ltd, England). The GAX substrate was dissolved under the same conditions as during the enzyme hydrolysis experiments, but without the addition of enzyme (1 mg mL^-1^ in 50 mM citrate buffer pH 6 at 40°C for 16 hours under agitation). The number and volume distribution (in %) were recorded as an average of 19 scans in triplicate (Figure S3). A refractive index increment (d*n*d*c*) of 0.133 mL g^-1^ for arabinoxylan was used for the DLS calculations.

(a) (b)

Figure S3 DLS analysis of the GAX substrate.

As can be observed for the number distribution, molecular solubility was completely achieved for the GAX substrate under these experimental conditions, providing an average hydrodynamic radius of 8 nm. Careful observation of the volume distribution offers an indication of the presence of minimal substrate aggregation, with two small peaks at 20nm and 200 nm. However, the relative abundance of these aggregation peaks corresponds to less than 1 % of the total substrate volume. The volume distribution is directly related to the number distribution by a power of 10^3^, which indicates again that the number of aggregated molecules was negligible.

### Quantification of the mono- and oligosaccharides released by the enzyme combinations

The reaction products from the single- and multi-enzyme reactions were quantified from the HPAEC-PAD chromatograms (main text, Figure 2) by comparing peak areas to those of standards standards of known concentrations. This quantification allows calculating the total monosaccharide release (as % wt of the dry substrate) presented in Table 2 of the main text.


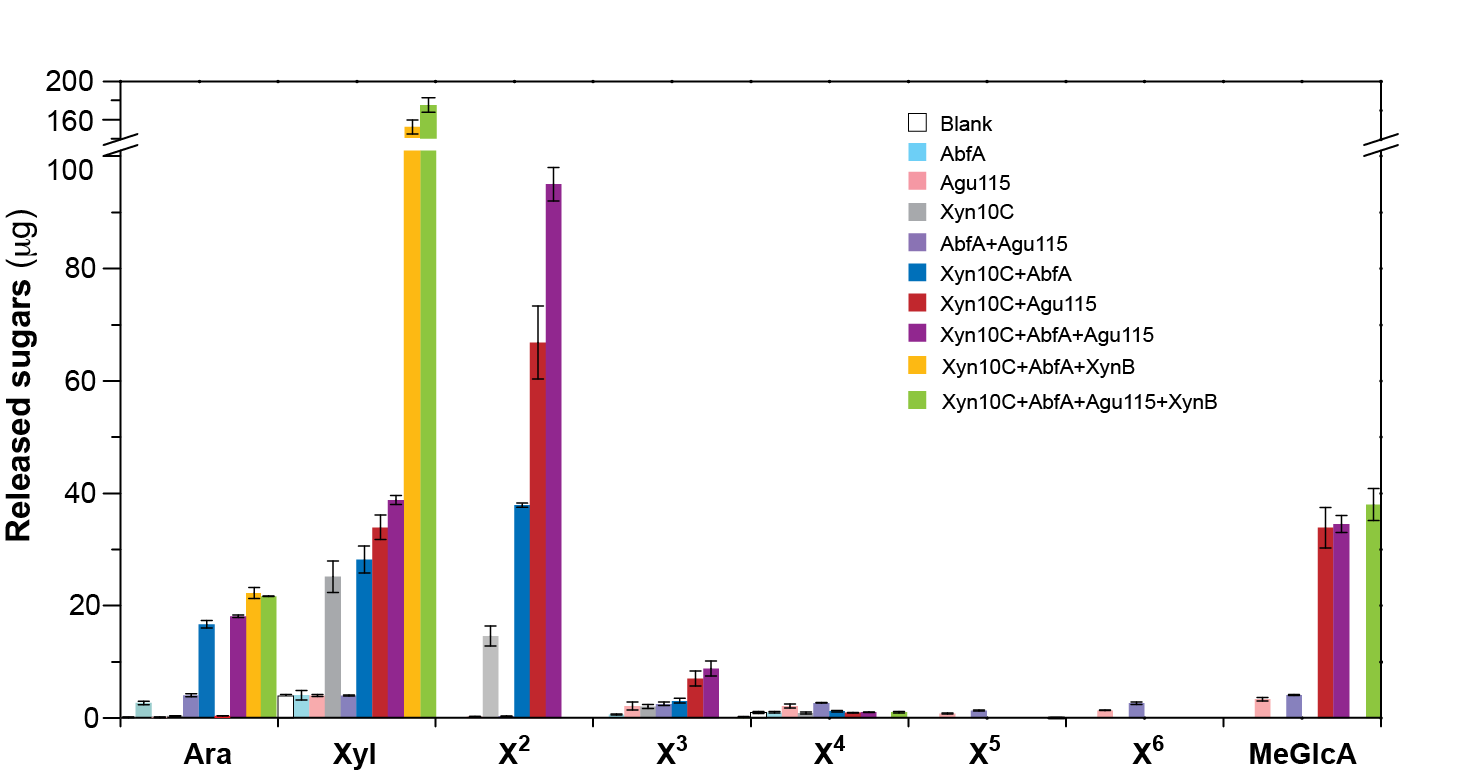


Figure S4 Quantification of the monosaccharides and linear xylo-oligosaccharides (XOs) released by each enzyme/enzyme combination.

## References

[1] P. Klason. Framställning af rent lignin ur granved och denna

sednares kemiska sammansättning. Teknisk Tidskrift 23:55–56 (1893)

[2] Tappi standard T 222 om-02. Acid-insoluble lignin in wood

and pulp, in 200-2003 TAPPI Test Methods, Tappi Press, Atlanta, GA, USA (2002).

[3] S Willför, A Pranovich, T Tamminen, J Puls, C Laine, A Suurnäkki, B Saake, K Uotila, H Simolin, J Hemming, B Holmbom. Carbohydrate analysis of plant materials with uronic acid-containing polysaccharides‚ A comparison between different hydrolysis and subsequent chromatographic analytical techniques. Industrial Crops and Products 29, 571, (2009).

[4] M. W. Davis. A Rapid Modified Method for Compositional Carbohydrate Analysis of Lignocellulosics by High pH Anion-Exchange Chromatography with Pulsed Amperometric Detection (HPAEC/PAD). Journal of Wood Chemistry and Technology, 18, 2, 235-252 (1998).
